# Emerging roles of ferroptosis in male reproductive diseases

**DOI:** 10.1038/s41420-023-01665-x

**Published:** 2023-09-28

**Authors:** Wenzheng Yuan, Zhibin Sun, Guojie Ji, Huanhuan Hu

**Affiliations:** 1 Key Laboratory of Fertility Preservation, School of Life Sciences and Technologies, Sanquan College of Xinxiang Medical University, Xinxiang, 453003 Henan Province PR China; 2 Institute of Life Sciences, China Medical University, Shenyang, 110122 Liaoning Province PR China

**Keywords:** Cell biology, Genetics

## Abstract

Ferroptosis is a type of programmed cell death mediated by iron-dependent lipid peroxidation that leads to excessive lipid peroxidation in different cells. Ferroptosis is distinct from other forms of cell death and is associated with various diseases. Iron is essential for spermatogenesis and male reproductive function. Therefore, it is not surprising that new evidence supports the role of ferroptosis in testicular injury. Although the molecular mechanism by which ferroptosis induces disease is unknown, several genes and pathways associated with ferroptosis have been linked to testicular dysfunction. In this review, we discuss iron metabolism, ferroptosis, and related regulatory pathways. In addition, we analyze the endogenous and exogenous factors of ferroptosis in terms of iron metabolism and testicular dysfunction, as well as summarize the relationship between ferroptosis and male reproductive dysfunction. Finally, we discuss potential strategies to target ferroptosis for treating male reproductive diseases and provide new directions for preventing male reproductive diseases.

## Facts

- Ferroptosis is a form of cell death that plays an essential role in various diseases.
- More and more evidence showed that ferroptosis is related to male reproductive diseases in recent years.
- Ferroptosis may be a new target direction for the prevention and treatment of male reproductive diseases.

## Open questions

- What is the progression of systemic iron metabolism?
- What physiological role does iron play in testis?
- What causes ferroptosis to occur and develop in testicular diseases? Is there a unique mechanism or signaling pathway?
- Can we develop more selective means to activate or inhibit ferroptosis either with small molecules or genetic manipulation for treating or reducing testicular diseases?

## Introduction

Infertility is a global health problem, with approximately 15% of couples suffering from infertility. The World Health Organization predicts that infertility will become the third most prevalent disease in the twenty-first century after cancer and cardiovascular disease [1]. Male infertility is caused by many risk factors such as varicocele, genital tract infection, and poor lifestyle habits [2, 3]. The incidence of infertility is increasing annually, and the infertility trend is observed in younger men. The testis is the site for the production of androgens and spermatozoa. Iron is an important metal element involved in the regulation of male reproductive function, and it has two roles in the testis [4]. First, sufficient iron is necessary to maintain testosterone synthesis and spermatogenesis [5], and second, excess iron affects sperm quality by inducing oxidative stress and lipid peroxidation, thus leading to male reproductive dysfunction [6, 7]. A recent study found that exposure of the testis to certain toxicants and risk factors, such as cadmium, busulfan, and smoking, can induce ferroptosis, suggesting targeted inhibition of ferroptosis may provide a strategy for protecting the testis from certain diseases [8].

Ferroptosis is a type of non-apoptotic cell death that can be triggered by iron accumulation and lipid peroxidation [9, 10]. Ferroptosis is different from other types of cell death [4]. In terms of morphology, the main features of ferroptosis are mitochondrial atrophy, condensed mitochondrial membrane density, and mitochondrial cristae loss. From a biochemical perspective, ferroptosis is induced by the accumulation of iron and the increase in reactive oxygen species (ROS) produced by lipid peroxidation. Ferroptosis is involved in various pathological states, including neurodegeneration, cancerization, ulcerative colitis, and ischemia-reperfusion injury of the kidney and liver, and it may play a key role in the development of certain diseases [11, 12]. This new evidence supports the role of ferroptosis in male reproductive diseases [13]. Targeting ferroptosis may provide a promising strategy for treating patients with reproductive dysfunction.

New evidence suggests that ferroptosis plays a key role in the pathophysiology of the testis. This information may provide new insights for treating certain reproductive diseases. Herein, we discuss the physiological significance of iron in male reproductive function in terms of iron metabolism. On this basis, the mechanisms of ferroptosis in various male reproductive diseases are analyzed and the presumed targets of ferroptosis in testicular injury are summarized. Lastly, this review article provides new insights for treating ferroptosis in the testis.

## Systemic iron circulation and regulation

**Fig. 1:**
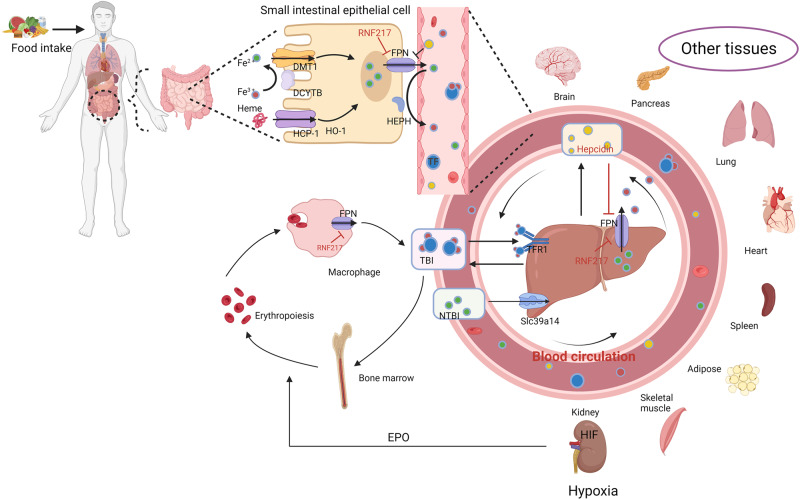
Systemic iron sources and metabolism. Dietary iron consists of Fe^3+^ and heme iron. Fe^3+^ is reduced to Fe^2+^ by DCYTB and then transported to intestinal cells by DMT1, whereas heme iron is transported to intestinal cells by HCP-1 and degraded by HO1 to produce Fe^2+^ in intestinal cells. Ferrous iron is released into capillaries via FPN (a unique iron exporter), where it binds to transferrin (TF) through oxidation of HEPN or CP. Finally, iron participates in the iron cycle through the TF-TFR1 pathway or SLC39A14-mediated NTBI. Macrophages degrade aged red blood cells to recycle iron, which represents another important source of iron for the body. EPO released by the kidney activates the HIF signaling pathway to promote erythrocyte production. The liver also plays an important role in iron regulation. TF and hepcidin are secreted by the liver to regulate iron homeostasis and used by the liver and other tissues. In addition to hepcidin, RNF217 maintains iron homeostasis by promoting the degradation of FPN. CP ceruloplasmin, DCYTB duodenal cytochrome b, DMT1 divalent metal transporter 1, EPO erythropoietin, FPN ferroportin, HCP-1 heme carrier protein 1, HEPH hephaestin, HIF hypoxia induced factor, HO1 heme oxygenase 1, NTBI non-transferrin-bound iron, RBCs red blood cells, TBI transferrin-bound iron, TFR1 transferrin receptor 1.

Iron, an essential metal element, is involved in various physiological processes such as DNA synthesis and repair, cellular metabolism, and oxygen transport [14]. The human body absorbs, transports, circulates, stores, and excretes iron. Iron homeostasis is strictly regulated to prevent iron deficiency and iron overload, which can cause various pathological states [15]. The main sources of iron in the human body can be divided into exogenous iron and endogenous iron [16]. Dietary iron, also known as exogenous iron, is absorbed mainly in the duodenum and upper jejunum, and this accounts for one-third of the human body’s iron intake. Iron in food exists as heme iron and non-heme iron, and the former is absorbed at a higher rate than the latter. Furthermore, the two types of iron enter the iron cycle through different mechanisms (Fig. 1). The second largest source of iron in the human body is the iron released by red blood cells, also known as endogenous iron, which accounts for two-thirds of the human body’s iron intake. Endogenous iron is released by aged or destroyed red blood cells, most of which is reused by the human body. The homeostasis of iron is also maintained through the regulation of iron absorption, as 1 to 2 mg of iron is lost daily due to skin desquamation, mucous cell sloughing, and blood loss, and the human body has no mechanism to actively remove iron [17]. The liver also plays an important role in regulating serum iron levels [18]. Excess iron is stored in liver cells as transferrin (TF)-bound iron or non-TF-bound iron. In addition, the liver secretes TF and hepcidin, which enter the circulatory system and travel to other tissues to maintain iron homeostasis. Jiang et al. [19] identified ring finger protein 217 as a novel E3 ligase mediating the degradation of ferroportin (FPN), which controls iron output alone or together with hepcidin.

## Driving and inhibiting ferroptotic mechanisms

**Fig. 2:**
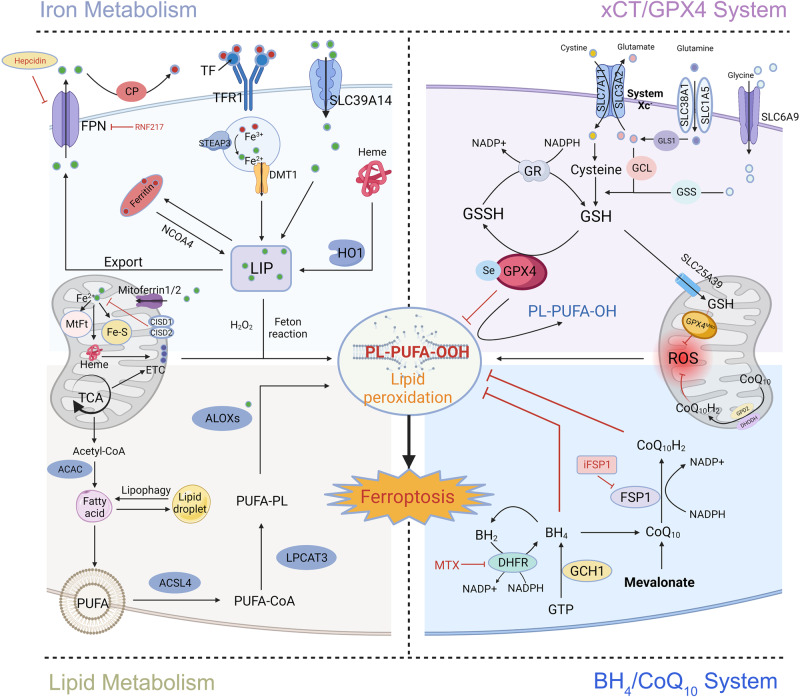
Mechanism of ferroptotic regulation. Excessive ferrous iron in cells is the direct cause of ferroptosis. An imbalance of iron homeostasis leads to ferroptosis. TBI and NTBI import exogenous iron into the intracellular, unstable iron pool via TF-TFR1 and SLC39A14. Ferritin stores iron and prevents excess ferrous iron from triggering oxidative stress through the Fenton reaction. Cellular iron is exported via FPN. In addition, HO1 oxidizes heme to Fe^2+^ and increases intracellular iron levels. Therefore, excessive iron input, blocked iron output, and NCOA4-mediated ferritin autophagy can increase intracellular iron levels, thereby increasing the susceptibility to ferroptosis. Iron also accelerates the lipid peroxidation of membrane phospholipids. The xCT/GPX4 system and the BH_4_/CoQ_10_ system eliminate the lipid peroxidation of membrane phospholipids and repair the damage of cell membranes. In addition, iron metabolism and the antioxidant system in mitochondria are involved in anti-ferroptosis. ACAC acetyl CoA carboxylase, ACSL4 acyl-CoA synthetase long-chain family member 4, ALOXs Arachidonate lipoxygenases, BH_2_ 7, 8-dihydrobiopterin, BH_4_ tetrahydrobiopterin, CoQ_10_H_2_ ubiquinol, CP ceruloplasmin, DHFR dihydrofolate reductase, DHODH dihydroorotate dehydrogenase, DMT1 divalent metal transporter1, ETC electron transport chain, Fe-S iron-sulfur clusters, FPN Ferroportin, FSP1 ferroptosis suppressor protein 1, GCH1 guanosine triphosphate cyclohydrolase 1, GCL γ-Glu-Cys ligase, GLS1 glutaminase 1, GPD2 glycerol-3-phosphate dehydrogenase 2, GPX4 glutathione peroxidase 4, GPX4^mito^ mitochondrial GPX4, GR glutathione reductase, GSH glutathione, GSS glutathione synthetase, GSSH glutathione disulfide, HO1 heme oxygenase 1, LIP labile iron pool, LPCAT3 lysophosphatidylcholine acyltransferase 3, FtMt mitochondrial ferritin, MTX methotrexate, NAC N-Acetyl cysteine, NCOA4 nuclear receptor coactivator 4, PUFA polyunsaturated fatty acid, ROS reactive oxygen species, STEAP3 six-transmembrane epithelial antigen of prostate 3, TCA tricarboxylic acid, TF transferrin, TFR1 transferrin receptor 1.

Ferroptosis, a type of non-apoptotic cell death, is characterized by three main processes that affect the sensitivity of cells to ferroptosis: (1) an increase in the content of free iron that generates ROS through the Fenton reaction, (2) a depletion of the antioxidant glutathione (GSH) and a loss of glutathione peroxidase 4 (GPX4) activity, and (3) an accumulation of lipid peroxidation products and a disruption of cell membranes [12]. With increasing research activity, several metabolic pathways related to ferroptosis have been reported, including the tetrahydrobiopterin (BH_4_)/coenzyme Q10 (CoQ_10_) system and the dihydroorotate dehydrogenase (DHODH)/CoQ_10_ axis [20]. In the subsequent sections of this review article, we discuss the roles of these metabolic pathways in ferroptosis (Fig. 2).

### Imbalances in iron metabolism induce ferroptosis

A disruption of the balance between iron uptake, storage, and export increases intracellular ferrous iron levels and induces ferroptosis, and excessive ferrous iron accumulation in cells causes ferroptosis. Transferrin receptor 1 (TFR1) and solute carrier family 39 member 14 (SLC39A14; also known as ZIP14) pathways regulate the entry of endogenous TF-bound and non-TF-bound iron into cells, whereas exogenous TF/TFR1 return to the cell surface for reuse [21, 22]. If ferrous iron is not immediately used for cellular processes, then it is stored by ferritin, which consists of heavy and light chains; otherwise, the cytoplasmic membrane protein FPN exports ferrous iron from cells [23]. Therefore, increased iron uptake and decreased iron output can lead to iron overload and thus ferroptosis. In addition to the disruption in iron homeostasis, nuclear receptor coactivator 4-mediated ferritinophagy can lead to iron accumulation and ferroptosis [24]. Notably, heme oxygenase 1 (HO1), which has a dual role in regulating ferroptosis, oxidizes cellular heme to carbon monoxide, ferrous iron, and biliverdin [25]. First, under normal conditions, elevated HO1 expression attenuates oxidative stress to exert cytoprotective effects, and second, sustained HO1 upregulation releases ferrous iron to exacerbate oxidative stress, which leads to ROS accumulation through the Fenton reaction. The significance of HO1 in ferroptosis has been shown in various pathological states such as liver injury, testicular injury, and colitis in different animal models [26–28]. Thus, a disruption in iron homeostasis can cause different diseases by triggering ferroptosis.

### Lipid peroxidation induces ferroptosis

Lipid peroxidation is an indicator of ferroptosis, and peroxidation of polyunsaturated fatty acid (PUFA)-containing phospholipids in cell membranes is an important step of ferroptosis [29]. Polyunsaturated fatty acids, such as arachidonic acid and adrenic acid, destabilize the lipid bilayer through peroxidation, thus affecting membrane function [30]. These fatty acids are catalyzed by acyl-coenzyme A synthetase long chain family member 4 (ACSL4) and converted to PUFA-coenzyme A (CoA) [31]. ACSL4 plays an essential role in ferroptosis. Lysophosphatidylcholine acyltransferase 3 (LPCAT3), an ER membrane protein, preferentially catalyzes ACSL4-acylated PUFA-CoA. Studies have shown that the absence of LPCAT3 or ACSL4 enhances ferroptotic resistance and reduces lipid peroxidation caused by ferroptosis [32]. In addition, lipoxygenases (ALOXs) are non-heme, iron-dependent dioxygenases that promote lipid peroxidation of PUFAs. Specifically, ALOXs facilitate the oxidation of PUFA or PUFA-containing lipids, thus contributing to ferroptosis [33]. In humans, there are six ALOX genes, namely *ALOX15*, *ALOX15B*, *ALOX12*, *ALOX12B*, *ALOXE3*, and *ALOX5*. In addition, ALOXs can directly or indirectly promote lipid oxidation in cell membranes to increase the sensitivity of cells to ferroptosis, especially under the catalysis of ferrous iron [34, 35]. Phospholipid hydroperoxide is produced by the peroxidation of phospholipids in cell membranes, and the decomposition of phospholipid hydroperoxide produces malondialdehyde and 4-hydroxynonenal [36]. Lipid peroxidation and its products can affect membrane function and ultimately lead to cell death. Therefore, inhibiting lipid peroxidation may be a potential strategy for treating diseases caused by ferroptosis.

### xCT/GPX4 pathway inhibits ferroptosis

The core molecular mechanism of ferroptosis involves regulating the balance between oxidative stress and antioxidant defense [37]. Glutathione is a tripeptide protein comprised of cysteine, glutamic acid, and glycine residues. Cysteine is the rate-limiting precursor in GSH synthesis [38]. Most cells obtain cysteine from the cystine/glutamic acid reverse transporter system Xc-, which includes solute carrier family 7 member 11 (SLC7A11, also known as xCT) and solute carrier family 3 member 2 (SLC3A2, also known as CD98). The transporter system Xc- imports extracellular cystine, while it exports intracellular glutamate at a 1:1 ratio [38, 39]. The absorption of cystine by system Xc- is an important step in the synthesis of GSH, which together with GPX4 inhibits the peroxidation of lipids. Inhibition of system Xc- expression leads to decreased GSH production, thus affecting GPX4 activity and inducing ferroptosis [40, 41]. In addition, GPX4 can reduce complex lipid peroxides to simple alcohols. The xCT/GPX4 axis, a major inhibitory pathway of ferroptosis, plays an important role in various diseases caused by ferroptosis. Many classical ferroptosis inducers, such as erastin and RAS-selective lethal 3, can target the xCT/GPX4 axis, and these inducers can trigger ferroptosis in cancer cells, suggesting they hold promise in the treatment of cancer [40, 42]. Thus, the xCT/GPX4 axis protects cells from ferroptosis by inhibiting lipid peroxidation.

### BH_4_/CoQ_10_ system inhibits ferroptosis

Because lipid peroxidation promotes oxidative stress and destroys cell membranes, it is a major inducer of ferroptosis in cells. Iron accumulation also promotes oxidative stress, destroys cell membranes, and induces ferroptosis. The BH_4_/CoQ_10_ system is comprised of two components, namely the ferroptosis suppressor protein 1 (FSP1)/CoQ_10_ axis and the guanosine triphosphate cyclohydrolase 1 (GCH1)/BH_4_ axis, and both pathways have been shown to inhibit ferroptosis independent of the xCT/GPX4 system [37]. An early study reported a similarity between FSP1 and apoptosis-inducing factor mitochondria-associated 2, but this finding was disproved in a subsequent study [43]. Existing evidence indicates that FSP1 localizes to plasma membranes and lipid droplets, and its anti-ferroptotic properties are due to its association with plasma membranes and not with lipid droplets [44]. The anti-ferroptotic FSP1 mechanism depends on NAD(P)H dehydrogenase and its reducibility. In addition, the FSP1 protein contains an N-terminal myristylation sequence, and only myristylated FSP1 has anti-ferroptotic activity. Other studies have shown that FSP1 inhibits lipid peroxidation and ferroptosis by reducing CoQ_10_ to ubiquitinol (CoQ_10_H_2_) [44, 45]. For example, Doll et al. [45] found that overexpression of FSP1 in cells lacking GPX4 expression significantly reduces the production of specific phospholipids. Furthermore, cells lacking FSP1 expression show increased sensitivity to ferroptotic inducers, including GPX4 (ML162) and xCT [45]. The FSP1 inhibitor iFSP1 can also induce ferroptosis or sensitize cells to other ferroptotic inducers [46]. In conclusion, these findings suggest that FSP1 inhibits ferroptosis through crosstalk among various pathways.

Genome-wide CRISPR screening identified GCH1, a new ferroptotic inhibitor that is independent of the xCT/GPX4 system but dependent and independent of the FSP1-CoQ_10_ system, which scavenges lipid peroxyl radicals [47]. The anti-ferroptotic properties of BH_4_ mainly involve its ability to function as a powerful antioxidant and to mediate CoQ_10_ synthesis. Furthermore, GCH1 is a rate-limiting enzyme that is synthesized from BH_4_. Recycling of BH_4_ requires dihydrofolate reductase (DHFR), and if DHFR is inhibited, then BH_4_ cooperates with GPX4 to induce ferroptosis [48]. Treatment with 7,8-dihydrobiopterin (BH_2_) protects cells from ferroptosis by producing BH_4_ through DHFR [49]. In addition to its function as a powerful antioxidant, BH_4_ also functions in the synthesis of CoQ_10_, an antioxidant [20]. Although crosstalk between BH_4_/CoQ_10_ and xCT/GPX4 pathways, which reduces lipid peroxidation and ferroptosis, has been reported, further studies are needed to investigate the anti-ferroptotic properties of the BH_4_/CoQ_10_ system.

### Regulatory role of mitochondria in ferroptosis

Mitochondria, semi-autonomous organelles, have unique metabolic mechanisms that allow these organelles to resist ferroptosis [50, 51]. Increasing evidence indicates that mitochondrial injury is a hallmark of ferroptosis. Iron enters mitochondria through SLC25A37 (also known as mitoferrin 1) and SLC25A28 (also known as mitoferrin 2). Mitochondrial ferrous iron can mediate the synthesis of heme and iron-sulfur (Fe-S); otherwise, it can be stored by mitochondrial ferritin (FtMt). The Fe–S-binding proteins mitoNEET (also known as CISD1) and NAF1 (also known as CISD2), which have been shown to participate in mitochondrial iron transport, inhibit ferroptosis by protecting mitochondria from lipid peroxidation in cancer cells and acute kidney injury [52, 53].

The mitochondrial tricarboxylic acid cycle occurs in the mitochondrial matrix where it interacts with various metabolic pathways [54, 55]. In brief, acetyl-CoA produced by glucose transfers electrons to the electron transport chain through a series of redox reactions, thereby generating ATP through oxidative phosphorylation as well as ROS, which induce ferroptosis. In addition, the tricarboxylic acid cycle serves as a redox platform for integrating metabolic signals from glycolysis and amino acid catabolism to produce PUFAs, which also induce ferroptosis.

Lipid peroxidation induces ferroptosis through its destructive effects on cell membranes. The mitochondrial electron transport cofactor CoQ_10_ can resist lipid peroxidation through a series of reactions, which occur on the outer surface of the inner mitochondrial membrane. Furthermore, mitochondrial GPX4 (GPX4^mito^) cooperates with the dihydroorotate dehydrogenase/CoQ_10_H_2_ pathway and the glycerol-3-phosphate dehydrogenase 2 (GPD2)/CoQ_10_H_2_ pathway on the inner mitochondrial membrane to inhibit ferroptosis [56, 57] (Fig. 2).

### Other regulatory pathways in ferroptosis

Studies have shown that other organelles, such as the endoplasmic reticulum, the Golgi apparatus, lysosomes, and peroxisomes, are also involved in ferroptosis [58, 59]. Notably, in a recent study, Liang et al. [60] identified phospholipid-modifying enzymes Lysophospholipid acyltransferase 1 (also known as MBOAT1) and Lysophospholipid acyltransferase 2 (also known as MBOAT2) as ferroptosis suppressors through a whole-genome CRISPR activation screen. MBOAT1/2 inhibit ferroptosis by remodeling the cellular phospholipid profile, and strikingly, their ferroptosis surveillance function is independent of GPX4 and FSP1. MBOAT1 and MBOAT2 are respectively upregulated at the transcriptional level by sex hormone receptors (estrogen receptor and androgen receptor). However, further studies are needed to identify the relevant mechanisms of ferroptosis.

## The role of ferroptosis in the testis

### Iron metabolism in the testis

Spermatogenesis occurs in the testis. This organ is comprised of spermatogenic cells, Leydig cells, Sertoli cells, and small blood vessels. Spermatogenic cells divide and differentiate into spermatozoa, which are released from the testis and transported to the epididymis. Leydig cells secrete androgens, which maintain male secondary sexual characteristics. Sertoli cells support spermatogenic cells and facilitate spermatozoal release. During spermatogenesis, the blood-testis barrier regulates the entry and the exit of various substances. Not surprisingly, spermatogenesis is an iron-dependent process, and iron metabolism in testis is strictly regulated [61, 62].

**Fig. 3:**
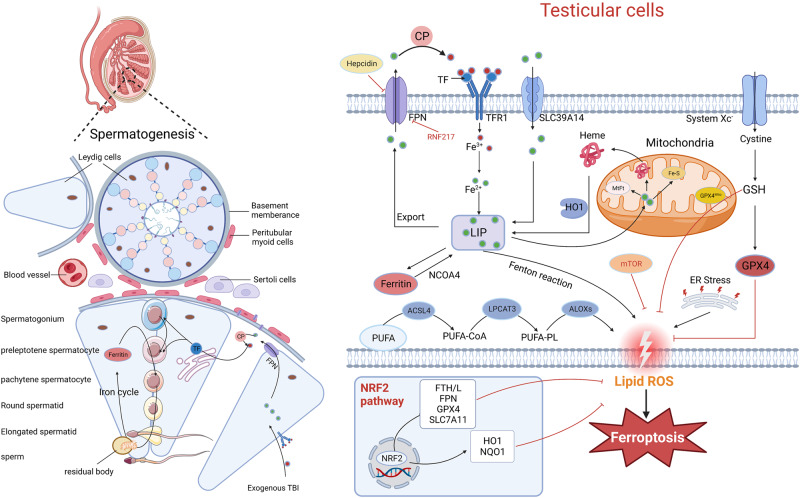
Testicular iron metabolism and ferroptosis. There are two forms of iron circulation in the seminiferous tubules (SFT). First, extracellular iron enters the Sertoli cells of the testis through the TF-TFR1 pathway, which is the main route of exogenous iron intake in Sertoli cells. As with other iron metabolic pathways, exogenous TBI is transported to Sertoli cells, and TBI is transported to endosomes. The FPN in Sertoli cells is responsible for iron release into the SFT gap. Sertoli cells synthesize and secrete CP, which oxidizes ferrous iron in the SFT gap to TF-bound iron. Iron output to the basement membrane of the SFT also requires TF, which is synthesized in the endoplasmic reticulum of Sertoli cells. Fe^3+^ binds to newly synthesized TF and is uptaken by spermatogenic cells into the circulation of iron. The second type of iron is derived from transport among spermatogenic cells at all levels. Iron is absorbed by spermatogonia and preleptotene spermatocytes through the TF-TFR1 pathway, and it is continuously transferred to round spermatids via different mechanisms. Eventually, round spermatids release some of the iron, which circulates to the apical compartment of Sertoli cells, and the rest of the iron is carried away by elongated spermatids. In addition, mitochondria in mature spermatozoa are phagocytosed by Sertoli cells in the form of residual bodies, and Sertoli cells store the iron in ferritin and return it to the iron cycle of primary spermatocytes, thereby completing the autonomous cycle of iron transport in the SFT. The loss of iron during spermatogenesis is compensated for by the absorption of exogenous iron. In addition to iron metabolism disorders, xCT/GPX4 system disturbances, mitochondrial dysfunction, and endoplasmic reticulum (ER) stress may increase the susceptibility to ferroptosis in the testis. Some transcription factors, such as NRF2, regulate genes related to ferroptosis under oxidative stress. ACSL4 acyl-CoA synthetase long-chain family member 4, ALOXs Arachidonate lipoxygenases, CP ceruloplasmin, Fe-S iron-sulfur clusters, FPN Ferroportin, FTH ferritin heavy chain, FTL ferritin light chain, GPX4 glutathione peroxidase 4, GPX4^mito^ mitochondrial GPX4, GSH glutathione, HO1 heme oxygenase 1, LIP labile iron pool, LPCAT3 lysophosphatidylcholine acyltransferase 3, FtMt mitochondrial ferritin, mTOR mammalian target of rapamycin, NRF2 nuclear factor erythroid 2-related factor 2, NCOA4 nuclear receptor coactivator 4, NQO1 quinone oxidoreductase 1, PUFA polyunsaturated fatty acid, ROS reactive oxygen species, TF transferrin, TFR1 transferrin receptor 1.

During spermatogenesis, iron released from spermatogonia and preleptotene spermatocytes is transferred to round spermatids, whereas iron released from round spermatids is transferred to Sertoli cells and stored by ferritin, which circulates to primary spermatocytes. Elongating spermatids uptake the remaining pool of iron, and the iron that is lost can be compensated by the absorption of exogenous iron. Leichtmann-Bardoogo et al. [61] found that peritubular myoid cells, Leydig cells, and Sertoli cells are the major storage sites of cytoplasmic ferritin in the testis and proposed the concept of compartmentalization of iron metabolism proteins to explain the maintenance of spermatogenesis (Fig. 3).

### Iron overload triggers ferroptosis in the testis

Iron overload is an important characteristic of ferroptosis [63]. Two general types of iron overload have been described. Primary iron overload is caused by genetic disorders that dysregulate the absorption of dietary iron, whereas secondary iron overload develops due to repeated blood transfusions, drug-induced toxicity, or excess consumption of iron [64]. Hereditary hemochromatosis, one of the most common genetic disorders in individuals of European descent, is characterized by significantly increased absorption of dietary iron. Excess iron accumulates because of the lack of an effective excretory mechanism, which leads to toxic effects. Clinical complications, such as liver cirrhosis, hepatocellular carcinoma, diabetes, cardiomyopathy, hypogonadism, arthropathy, and osteoporosis, may occur [65]. *Hfe* is the gene mutated in the most prevalent form of hereditary hemochromatosis. *Hfe* mutations cause constitutively low levels of the iron-regulating hormone hepcidin and systemic iron overload [66]. However, in *Hfe* knockout mice, a wide range of mutations can cause various degrees of iron overload in several organs, including the liver and heart [65]. Because of the unique mechanism of iron regulation in the testis, no significant iron overload was found in this organ [61]. However, it is worth noting that iron accumulation at the hypothalamic-pituitary level may impair Gonadotropin-releasing hormone neurons and/or pituitary gonadotroph cells and lead to hypogonadotropic hypogonadism, which is characterized by low levels of gonadotropins (FSH and LH) and testosterone, thus indirectly affecting testicular function [67, 68]. Whatever the underlying genetic cause that leads to iron overload, clinical manifestations and organ damage depend on the amount and the rate of iron accumulation in tissues and the different capacity of cells and tissues to cope with the iron-induced oxidative damage.

Therefore, the main cause of iron overload in the testis is the degradation of the reservoir or the obstruction of the outlet, which is induced by secondary iron overload [8, 13]. For example, abnormal iron levels are observed in semen specimens of smoking patients with asthenospermia, suggesting a role for ferroptosis in male reproductive dysfunction. In addition, testicular injury caused by cadmium and busulfan exposure is associated with decreased FPN expression. After treatment with deferoxamine (DFO, an iron chelator), the abnormal iron content and sperm defects can be reversed [69, 70]. Furthermore, Li et al. [71] confirmed that ferroptosis is a pervasive and dynamic type of cell death induced by oxygen-glucose deprivation and reoxygenation injury in Sertoli cells. Cell death was blocked by the ROS inhibitor N-acetylcysteine, as well as lipid peroxidation inhibitors liproxstatin‑1 and DFO; however, inhibitors of apoptosis, necrosis, or autophagy had no effect. Increasing evidence suggests that iron overload is an important feature of various male reproductive diseases (Table 1), indicating an indispensable role for ferroptosis in the male reproductive system. However, given the lack of robust biomarkers for measuring the levels of ferroptosis in patients, the association between ferroptosis and these diseases remains poorly understood. This will be an important research area worthy of study in the future.

**Table 1:** Inducers of testicular ferroptosis.

Factor/reagent	Categories	Mechanism	Ref.
Busulfan	Drug	Downregulated NRF2ˎGPX4 and FPN expression	[70]
Tripterygium wilfordii polyglycoside tablet	Drug	Inhibit NRF2/xCT/GPX4 axis and FPN espression	[118, 119]
Ischemia-reperfusion	Testicular torsion	Downregulated NRF2ˎGPX4 and FPN expression	[71]
Scrotal hyperthermia	High temperature exposure	Increase ACSL4 expression	[107]
Smoking (nicotine)	Habit	Downregulated NRF2 and GPX4 expression	[82, 108]
2.5-micron particulate matter	Environmental factor	Depletion of GSH and reduce GPX4 expression	[83, 110]
DEHP	Environmental factor	Increase TFR1 and HO1 expression Reduce GPX4 level	[27, 126, 135]
BisphenolA	Environmental factor	Downregulate NRF2/HO1 and xCT/GPX4 pathwayˎActivate ACSL4 expression and ROS accumulation	[111, 112]
Arsenite	Heavy mental	Downregulate xCT/GPX4 pathwayˎInduce ROS release and increase iron levels	[84]
Cdmium	Heavy mental	Reduce FTHˎFPN and xCT expressionˎDownregulate NRF2/HO1 pathway and enhance lipid peroxidation	[69, 115]
Hexavalent chromium	Heavy mental	Inhibit xCT/GPX4 and mTOR	[99]
Copper	Heavy mental	Regulate AMPK/mTOR pathway	[100]
Zinc	Heavy mental	downregulate levels of SLC7A11, GPX4, and ferritin, and upregulate levels of TFR1, TF	[98]
Deoxynivalenol	Biotic factor	Inhibit NRF2/xCT/GPX4 axis	[114]
Zearalenone	Biotic factor	Inhibit NRF2/xCT/GPX4 axis	[113]
HT-2 toxin	Biotic factor	Inhibit xCT/GPX4 axis and increase ACSL4 expression	[103]

### Lipid metabolism and ferroptosis in the testis

Lipid peroxidation is triggered by a reaction involving lipids with ROS. Spermatozoal membranes are rich in PUFAs. In addition, spermatozoa lack cytoplasmic ROS scavengers, indicating spermatozoa are more susceptible to lipid peroxidation than other testicular cells. Excessive ROS production can interfere with DNA synthesis and repair, which affects germ cell function [12, 13]. Before the introduction of the concept of ferroptosis in 2012, lipid peroxidation was believed to reduce the fluidity of spermatozoal membranes, affect the quality of spermatozoa, and disrupt membrane fusion during sperm-egg binding [72, 73]. Another study has shown that developed germ cells, such as spermatozoa, are more susceptible to oxidative stress and ferroptosis than undeveloped germ cells [74]. In the mouse testis, the interaction of ACSL4 with ALOX15 in round spermatids can increase the susceptibility of these cells to ferroptosis.

Taken collectively, these findings indicate that ferroptosis is accompanied by oxidative stress and lipid peroxidation in testicular injury, which supports the hypothesis that lipid peroxidation plays a role in both the development and the severity of testicular dysfunction. Therefore, the elimination of lipid hydroperoxides can inhibit lipid peroxidation, oxidative stress, and ferroptosis in germ cells.

### Glutathione metabolism and ferroptosis in the testis

SLC7A11 is a major transporter of system Xc−. Cystine enters cells through system Xc− and is reduced to cysteine for GSH synthesis. Studies have shown that SLC7A11 is highly expressed by cells of the testis, such as Sertoli cells, cells of the epididymis [75]. The decreased reproductive performance of *SLC7A11* knockout male mice was supported by a high proportion of immature spermatozoa in the cauda epididymis, although there were no significant differences in cysteine and GSH levels in the testis compared to controls, suggesting GSH synthesized by xCT is considered to be an important antioxidant [76]. Reactive oxygen species produced during spermatozoal maturation can be eliminated by antioxidant enzymes in the semen, indicating the importance of antioxidants in maintaining male reproductive function. GPX4 requires GSH as a cofactor to inhibit lipid peroxidation, and GPX4 has gained research momentum because of its important role in maintaining spermatogenesis [77]. GPX4 is a selenium protein expressed by cells of various organs, and its synthesis and activity are affected by selenium and GSH levels [78, 79]. Studies have shown treatment of germ cells with selenium can increase GPX4 expression, maintain spermatozoal motility, and increase germ cell proliferation, indicating the xCT/GPX4 system plays a role in male infertility [80, 81]. In another study, sperm specimens from smoking patients showed decreased spermatozoal motility and decreased GPX4 expression [82]. In addition, reduced SLC7A11 and GPX4 expression was reported in testicular injury induced by 2.5-micron particulate matter and arsenite [83, 84]. Elevated iron levels were associated with these causes of testicular injury. Spermatocyte-specific *Gpx4* knockout mice were infertile [85]. Taken collectively, these findings illustrate the important role of the xCT/GPX4 axis in male reproductive function.

### Mitochondria and ferroptosis in the testis

Mitochondria are the powerhouses and the sources of ROS production in eukaryotic cells [50]. In the testis, mitochondria play important roles in supporting spermatogenesis, promoting sperm motility, and regulating apoptosis [86]. Studies have reported that disruptions in mitochondrial structure, function, and dynamics are closely related to male infertility. New evidence suggests that mitochondria play a key role in ferroptosis [87]. Mitochondrial ferritin, which is expressed by spermatozoa and Leydig cells, protects mitochondria from oxidative stress induced by the Fenton reaction [88]. In addition, the number of mitochondria increase from primary spermatocytes to round spermatids, a process that requires iron for mitochondrial iron–sulfur clusters and heme synthesis. In the final steps of spermatid maturation, mitochondria are shed into residual bodies for recycling to primary spermatocytes to participate in the iron cycle [8, 13, 61].

In addition to unique metabolic processes, mitochondria contain GPX4, which like GPX4 in the cytoplasm, inhibits lipid peroxidation and ferroptosis. GPX4^mito^, the main selenoenzyme in the testes, is essential for spermatogenesis [89]. Systemic *Gpx4* knockout is embryonically lethal, whereas conditional *Gpx4* knockout promotes disorders of the brain, liver, vascular system, hematopoietic system, and immune system [90]. Manuela et al. [91] found that mitochondrial *Gpx4* deficiency can lead to male infertility.

### NRF2 pathway and ferroptosis in the testis

Nuclear factor erythroid 2-related factor 2 (NRF2) is a transcription factor that regulates antioxidant reactions, and its antioxidant effects lie in its ability to induce the expression of downstream antioxidant genes. In brief, NRF2 detaches from Kelch-like ECH-associated protein 1 and translocates to the nucleus, where it activates the expression of downstream target genes containing antioxidant responsive elements. Many downstream target genes of NRF2, such as HO1, SLC7A11, FTH/L, and FPN, are related to the regulation of ferroptosis, suggesting NRF2 is a key negative regulator of ferroptosis [92]. A significant decrease in sperm quality was found in *Nrf2* knockout mice [93]. In a model of oligospermia, the expression of NRF2, GPX4, and FPN was significantly downregulated by busulfan exposure, and ferroptotic inhibitors reversed these changes in expression. In addition, the NRF2 agonist sulforaphane upregulated GPX4 and FPN expression and significantly improved sperm concentration and motility [70, 94]. These findings indicate that ferroptosis is strongly associated with male reproductive disorders, specifically the death of different testicular cells due to oxidative damage. Therefore, targeting the NRF2 pathway is an important strategy for treating male infertility.

## Ferroptosis-related biological responses in testicular injury

**Fig. 4:**
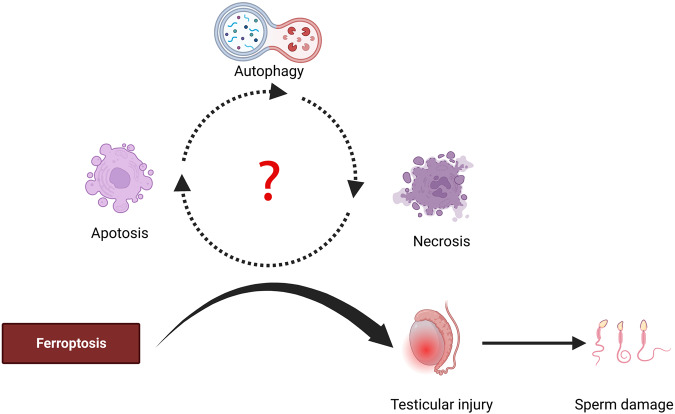
Role of ferroptosis in testicular diseases. Ferroptosis plays an important role in the progression of testicular diseases. In some cases, ferroptosis, autophagy, apoptosis, and necrosis work together to promote testicular diseases in a synergistic or a sequential manner. However, the relationship between these types of cell death and testicular diseases requires further research.

As an iron-dependent form of non-apoptotic cell death, ferroptosis is distinct from other types of cell death (apoptosis, necrosis, and autophagy). However, there is increasing evidence that several modes of cell death co-exist in testicular diseases, which also implies that ferroptosis is associated with several modes of cell death in testicular diseases. In the subsequent sections, we discuss the roles and differences between ferroptosis and other forms of cell death in testicular diseases (Fig. 4).

### Autophagy

Autophagy, the process of cell content degradation, is a very important biological phenomenon involved in the development and growth of organisms [95, 96]. Autophagy-dependent cell death is closely regulated. Under the regulation of autophagy-related genes, eukaryotic cells use lysosomes to degrade their own cytoplasmic proteins and damaged organelles, to remove damaged or harmful components, and to maintain the dynamic balance between nutrition and energy.

However, excessive autophagy may induce ferroptosis. Ferritinophagy mediated by NCOA4 promotes ferritin degradation and iron release, thereby increasing free iron content and further inducing ferroptosis [97]. In a zinc-induced porcine model of testicular dysfunction, zinc induces ferroptosis by regulating mitochondrial autophagy [98]. Hexavalent chromium also causes testicular autophagy and ferroptosis in rats [99]. In addition, classical ferroptosis activators (such as ML-210) or inhibitors (such as ferrostatin-1, Fer-1) modulate intracellular autophagic flux in testicular injury. Similarly, an autophagy inhibitor, 3-methyladenine, has been shown to alleviate testicular ferroptosis [98, 99]. Two sides of autophagy have been revealed in the study of testicular injury induced by copper sulfate. First, autophagy plays a protective role by inhibiting oxidative damage and apoptosis in CuSO_4_-induced testicular injury and spermatogenesis disruption, and second, autophagy plays an adverse role by promoting ferroptosis, thus aggravating the toxicity caused by CuSO_4_ [100]. At present, the relationship between autophagy and ferroptosis is controversial and requires further research.

In summary, there is a complex association between autophagy and ferroptosis. Ferroptosis is considered to be an autophagic process [101], and controlling autophagy during ferroptosis may be a therapeutic strategy for certain testicular diseases.

### Apoptosis

Apoptosis, the orderly death of cells that is autonomously controlled by genes, maintains the homeostasis of the environment. It is a process that has evolved and adapted to different environments. Apoptotic bodies are formed during apoptosis. Although there is no direct relationship between ferroptosis and apoptosis, the two processes often co-exist as shown in various models of testicular injury. CuSO_4_, cadmium, and HT-2 toxin can induce ferroptosis and apoptosis in the testis [69, 100, 102, 103]. The use of apoptosis inhibitors seems to have no effect on ferroptosis; however, apoptosis and ferroptosis can be regulated by controlling the production of ROS [103, 104], suggesting both processes may exert indirect effects and/or synergistic effects in certain injuries.

### Necrosis

Necrosis is the death of cells induced by serious pathological factors; this type of cell death is also known as pathological cell death. Necrotic cells are characterized by swelling, deformed and/or enlarged organelles, and finally cell rupture, which releases inclusions that can cause an inflammatory response. With continued research on necrosis, investigators have divided the process into necroptosis and pyroptosis [105]. The involvement of ferroptosis and necrosis in testicular injury induced by heat stress has been reported [106, 107]. In other cases, ferroptosis and necroptosis have been demonstrated to act in a synergic or a sequential manner. However, there is no evidence to prove the role of necrosis and ferroptosis in the current study of testicular injury.

As mentioned above, there may be a close association among ferroptosis, autophagy, apoptosis, and necrosis in testicular diseases. However, testicular diseases are very complex, and the relationship between ferroptosis and other forms of cell death remains to be studied.

## Ferroptosis in male reproductive dysfunction

**Fig. 5:**
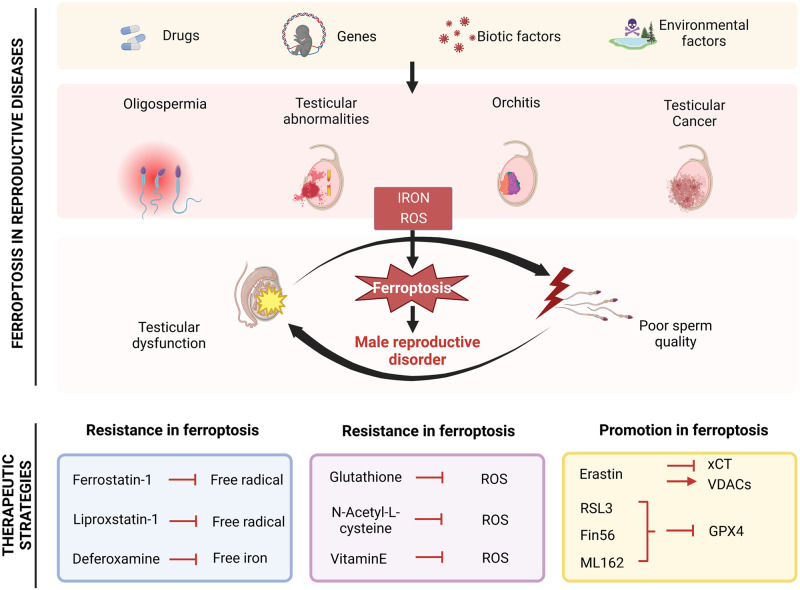
Strategies for targeting ferroptosis in the treatment of testicular diseases. Exposure to risk factors (such as harmful biochemical substances, drugs, and inadequate living conditions) can lead to testicular injury, as well as dysregulation of ROS production and iron metabolism in the testis. In addition, targeting ferroptosis can alleviate reproductive toxicity, including seminiferous tubule damage, mitochondrial dysfunction, and low sperm quality. Ferroptosis plays a complex role in testicular disease and may offer a new direction in the treatment of testicular diseases by targeting ferroptosis. GPX4 glutathione peroxidase 4, ROS reactive oxygen species, VDACs voltage-dependent anion channels, xCT solute carrier family 7 member 11.

A growing number of studies have demonstrated the relationship between ferroptosis and testicular diseases in vivo and in vitro. Studies of ferroptosis in the testis have suggested that endogenous and exogenous factors may be to blame (Fig. 5). However, the regulatory mechanisms of ferroptosis in testicular injury remain unclear, and they should be explored in various models.

### Testicular ferroptosis due to unhealthy lifestyle factors

Poor lifestyle habits, such as smoking, drinking, and insufficient sleeping, may affect spermatozoal quality and cause male infertility. A population-based study has shown that sperm motility and forward movement are significantly decreased in heavy smokers compared to non-smokers, and oxidative stress and iron levels are elevated [82]. Nicotine is the main harmful substance in cigarettes, and nicotine affects male reproductive function by disrupting blood-testis barrier dynamics [108]. The study also revealed that NRF2 mediates nicotine-induced ferroptosis. Fer-1, a ferroptotic inhibitor, reverses testicular injury, suggesting smoking induces ferroptosis. Studies in obese rats have reported increased iron and hepcidin levels, as well as defects in mitochondria and endoplasmic reticula in the testis, which have been shown to be involved in ferroptosis [109]. However, whether ferroptosis is involved in obesity-induced ferroptosis in the testis is unknown.

### Testicular ferroptosis caused by environmental factors

Societal changes have improved the lives of many, although certain advancements may adversely affect human health. For example, 2.5-micron particulate matter is an environmental pollutant that has been demonstrated to induce iron overload and lipid peroxidation, leading to male reproductive dysfunction [83, 110]. Bisphenol A and DEHP, commonly used chemicals in the plastics industry, have also been shown to induce ferroptosis in the testis [27, 111]. Tetramethyl bisphenol A, a bisphenol analog, is used as a fire retardant, and it inhibits testosterone synthesis in late puberty by inducing ferroptosis in Leydig cells [112]. In the food chain, zearalenone and deoxynivalenol are mycotoxins that induce male reproductive toxicity. Studies have shown that zearalenone significantly reduces spermatozoal motility and concentration in mice and destroys germ cell organization within the seminiferous tubules, possibly due to an imbalance of the antioxidant system [113, 114]. Both mycotoxins increase lipid peroxidation in the testis by downregulating NRF2, SLC7A11, and GPX4 expression. A recent study has reported that HT-2 toxin, another mycotoxin, inhibits Leydig cell proliferation and testosterone secretion and induces ferroptosis and apoptosis through the accumulation of ROS, leading to lipid peroxidation [103].

### Testicular ferroptosis induced by heavy metals

The total heavy metal content in the environment caused by human activities has increased beyond the acceptable range. Cadmium is a widespread, highly toxic heavy metal that accumulates in certain organs, mainly in the kidney. This heavy metal has been shown to reduce male fertility [69, 115]. For example, in the pubertal mouse, cadmium disrupts iron metabolism and antioxidant signaling, triggers ferroptosis in spermatogonocytes, and impairs spermatogenesis and testicular development. Another study has shown that ferroptosis induced by hexavalent chromium and copper can explain the role of mammalian target of rapamycin-mediated autophagy [99, 100]. Arsenic is commonly found in the environment as arsenite, which induces lipid peroxidation and mitochondrial dysfunction in the testis [116]. Arsenite has been shown to induce oxidative stress, lipid peroxidation, mitochondrial dysfunction, and ferroptosis in the testis, indicating arsenite triggers testicular cell death by inducing ferroptosis [84]. A recent study has demonstrated that mitophagy also contributes to zinc-induced ferroptosis in porcine testis cells, providing new insights into heavy metal toxicology [98].

### Drug-induced testicular ferroptosis

Many commonly used drugs can induce testicular injury. Reproductive toxicity induced by drugs can only be alleviated by drug withdrawal or dosage control. Busulfan is an anticancer drug that kills undeveloped germ cells, affects spermatozoal production, and induces male infertility, and the drug has been used to establish a model of oligospermia. Zhao et al. [117] found that ferroptosis induced by busulfan may be mediated by the inhibition of NRF2, GPX4, and FPN. *Tripterygium wilfordii*, a traditional Chinese medicine, is used to treat rheumatoid arthritis. However, the reproductive toxicity of *Tripterygium wilfordii* limits its clinical application, and its mechanism of reproductive toxicity is not fully understood [118]. Qin et al. [119] combined metabolomics with transcriptomics and found that ferroptosis was associated with *Tripterygium wilfordii*-induced testicular injury. Therefore, targeting ferroptosis is a potential strategy to prevent drug-induced testicular injury and male infertility.

### Other testicular diseases

Ferroptosis has been studied in other types of testicular injury (Table 1). Ischemia-reperfusion injury caused by testicular torsion induces Sertoli cell death, leading to male reproductive dysfunction [71]. Elevated iron and lipid ROS levels, as well as mitochondrial damage, are indicative of ferroptosis during ischemia-reperfusion injury. In addition, radiation affects reproductive function by causing antioxidant imbalances and disrupting iron metabolism in the testis [120–122]. However, it is unclear whether ferroptosis is involved in radiation-induced damage. Varicocele and scrotal hyperthermia have also been shown to associate with ferroptosis [107, 123, 124]. In a recent RNA-sequencing study, *Gpx4* expression in spermatozoa was downregulated in a model of experimental autoimmune orchitis [125]. However, the involvement of ferroptosis in autoimmune orchitis is unknown. As ferroptosis and testicular diseases continue to be investigated, new preventive strategies will be identified and tested in different clinical settings.

## Therapeutic strategies

Given the role of ferroptosis in the pathogenesis of testicular diseases, ferroptosis is a very promising therapeutic target for treating and preventing male reproductive diseases. In the subsequent sections, we discuss the molecules that can inhibit ferroptosis and the application of these molecules in models of testicular injury (Table 2).

**Table 2:** Summary of potential ferroptosis-targeted therapies in testicular diseases.

Drugs	Subjects	Mechanism	Ref.
Ferrostatin-1	Mice/Rats/Audults	Inhibits lipid peroxidation	[40, 69, 70, 74, 82–84, 113]
Liproxstain-1	Mice	Inhibits lipid peroxidation	[71]
Vitamin E (α-tocopherol)	Mice	Inhibits lipid peroxidation	[40]
Deferoxamine	Mice/Rats	Iron chelator	[69–71, 74, 83, 136, 137]
N-Acetyl cysteine	Mice	Recover intracellular cysteine	[71]
Melatonin	Sheep/mice	Regulates glucose-6-phosphate dehydrogenase/glutathione -dependent pathway	[103]
Sulforaphane	Mice	Activation of NRF2	[70]
Bone marrow mesenchymal stem cells transplantation	Rats	Alleviats ferroptosis and downregulates autophagy-associated proteins through the upregulation of AKT and mTOR phosphorylation	[99]
Guilu-Erxian-Glue	Rats	Activats the Keap1/Nrf2/GPX4 signaling pathway to resist ferroptosis	[118]
Alpha-lipoic acid	Rats	Activats NRF2/xCT/GPX4 axis	[123]
Glucagon-like peptide-1	Rats	antioxidant capacity	[138]
RSL3	Testicular cancer cells	Inhibition of GPX4	[133]

### Inhibitors of ferroptosis

Iron metabolism plays an important role in ferroptosis, and high iron levels have been shown to cause ferroptosis, suggesting iron chelation therapy may be an effective method to prevent ferroptosis. For example, the ferroptotic inhibitor DFO chelates iron and prevents lipid peroxidation of membrane lipids by limiting the Fenton reaction [9]. Presently, few iron chelators have been approved by the United States Food and Drug Administration, and few chelators are under study in clinical trials [9, 70]. Fer-1, a commonly used ferroptotic inhibitor that can eliminate free radicals and lipid peroxides, has been tested in various diseases, including testicular dysfunction and cancer [82, 93, 126]. However, clinical data of the effects of ferroptotic inhibitors are lacking [18]. The use of ferroptotic inhibitors may also be limited because of undesirable pharmacokinetic and pharmacodynamic characteristics.

### Antioxidants

In addition to iron chelators and ferroptotic inhibitors, antioxidants can eliminate ROS and activate other pathways to prevent ferroptosis, although this type of cell death is not entirely due to oxidative stress resulting from ROS toxicity. Ferroptosis involves the oxidation of specific types of lipids, suggesting antioxidants may reverse certain male reproductive disorders. The glutathione precursor N-acetyl cysteine is a widely used antioxidant, and studies have shown that vitamin E, Guilu Erxian Glue, and alpha lipoic acid can alleviate ferroptosis in the testis [40, 100, 118, 123]. The NRF2 inducer sulforaphane can improve spermatozoal concentration and quality in mice treated with busulfan [70]. Quercetin, GSH, vitamin C, vitamin B9, carotenoids, carnitines, polydatin, and taurine have also been reported to protect the testis from damage and improve semen quality, but their roles in ferroptosis have not been elucidated [127–130]. Therefore, further studies are needed.

### Targeting ferroptosis in testicular cancer

Testicular cancer is the most common malignancy in men aged 20 to 35 years. Most testicular tumors are malignant, and seminoma is the most common type of testicular tumor. The etiology of the disease is unknown, although it is believed that the pathogenesis is related to both inherited and acquired factors [131]. Testicular tumors are surgically resected, which is often followed by radiotherapy and chemotherapy. Chemotherapy drugs include cisplatin, bleomycin, doxorubicin, and cyclophosphamide. Interestingly, a recent study has shown that testicular cancer may be treated by targeting ferroptosis [132]. However, few studies have described the role of ferroptosis in testicular cancer [133, 134].

## Conclusions and perspectives

The role of ferroptosis in male reproductive dysfunction has been reported. Excess iron can induce testicular injury. Antioxidants or inhibition of ferroptosis with iron chelators may prevent and treat testicular diseases because inhibition of ferroptosis has been shown to significantly improve male reproductive function in various animal models. However, few clinical trials have been conducted to evaluate the therapeutic effects of ferroptotic inhibitors on male reproductive diseases. More population-based data are urgently needed to determine whether selective blocking of ferroptosis can reverse testicular dysfunction.

A growing body of evidence supports the important role of ferroptosis in male reproductive dysfunction. Although progress has been made in the field of ferroptosis, further studies are needed to determine whether ferroptotic inhibitors can reverse testicular dysfunction and restore male fertility. Several questions should be addressed in future research. First, ferroptosis often co-exists with other types of cell death such as apoptosis, necrosis, and autophagy. Inhibition of ferroptosis alone may not completely reverse testicular dysfunction caused by other types of cell death. Thus, the extent to which ferroptosis participates in testicular dysfunction still requires further research. Second, biomarkers for predicting ferroptosis, and thus male reproductive dysfunction should be identified. Third, strategies for treating testicular dysfunction caused by different factors should be developed. Fourth, specific targets of ferroptosis should be identified. Further research is needed to address these key issues and to improve our understanding of the role of ferroptosis in male reproductive dysfunction, which may provide new insights on the treatment and prevention of testicular diseases.
